# Rescue ross procedure and mitral valve repair on a low birth weight preterm neonate

**DOI:** 10.1111/jocs.16865

**Published:** 2022-08-21

**Authors:** Luigi Garufi, Angela di Candia, Francesco Bertelli, Alvise Guariento, Vladimiro L. Vida

**Affiliations:** ^1^ Department of Cardiac, Thoracic, and Vascular Sciences University of Padua Padova Italy; ^2^ Unit of Pediatric and Congenital Cardiology, Department for Women's and Children's Health, University Hospital of Padova University of Padua Padova Italy

**Keywords:** neonatal cardiac surgery, Ross procedure

## Abstract

Although mid‐ and long‐term outcomes after the Ross procedure for aortic valve disease have been increasingly improving over the years, this is still a rather challenging operation in neonates and small children. This is particularly true for patients with associated congenital heart defects and critical clinical conditions. Herein we describe the application of this procedure as a rescue operation in emergency circumstances in a low‐birth‐weight neonate with severe aortic stenosis, aortic regurgitation and mitral regurgitation after a previous aortic coartectomy.

**Abbreviations**
MVP: mitral valve plasty
PDA: patent ductus arteriosus
POD: postoperative day
RP: Ross procedure

## 1 INTRODUCTION

First described by Donald Nixon Ross^1^ in 1967, the Ross procedure is still the preferred option for performing aortic valve replacement in children and young adults.^2^ The substantial advantage of the pulmonary autograft is its potential for growth, repair, and adaptation as a living structure.

Nevertheless, recent series showed that operative mortality for infants weighing less than 3 kg is substantial especially in combination with other congenital cardiac anomalies.^3^

Here we describe a technical tour de force on a baby born with aortic valvulopathy, coarctation and mitral valve dysplasia.

## 2 CASE REPORT

A 30‐week gestational premature male neonate, weighing 1.538 kg, presented to our Department of Neonatal Pathology for hemodynamic instability caused by aortic stenosis with severe left ventricular dysfunction. The mean gradient was 70 mmHg. Intubation and surfactant administration were required due to respiratory failure with severe acidosis.

When the infant was 9 days of age, percutaneous balloon aortic valvuloplasty was performed with an echocardiogram showing moderate mitral regurgitation and a patent foramen ovale with a left‐to‐right shunt. One week later, another echocardiography revealed moderate aortic stenosis and aortic coarctation (likely initially masked by a patent ductus arteriosus [PDA]). The prostaglandin infusion was started immediately and after 5 days the patient underwent coartectomy and PDA ligation via left thoracotomy.

One month after coartectomy, severe hypotension occurred, and administration of epinephrine was required. Weaning of the baby from mechanical ventilation was not possible due to hemodynamic instability and an important edematous state worsened the clinical condition. Echocardiography showed massive mitral regurgitation and severe aortic steno‐insufficiency. The child then developed necrotizing enterocolitis (stage 2A according to the modified Bell staging criteria for necrotizing enterocolitis) and triple antibiotic therapy with ceftazidime, vancomycin, and metronidazole was started. At that point, echocardiography (Video 1) revealed a dilated left ventricle and a left mega‐atrium (17 mm in diameter, *z* score = +3.3) with jet‐to‐roof lesions. Massive mitral regurgitation was present due to prolapse of a posterior hypomobile leaflet and coaptation deficiency. The aortic valve appeared bicuspid and severely dysplastic with a diameter of 10 and 7 mm on the short and long axis, respectively. The pulmonary artery had a diameter of 8.6 mm (*z* score = +1.5). Severe aortic insufficiency with holosystolic outflow into the abdominal aorta and a median transvalvular gradient of 34 mmHg was identified on Doppler echocardiography.

**Figure 1:**
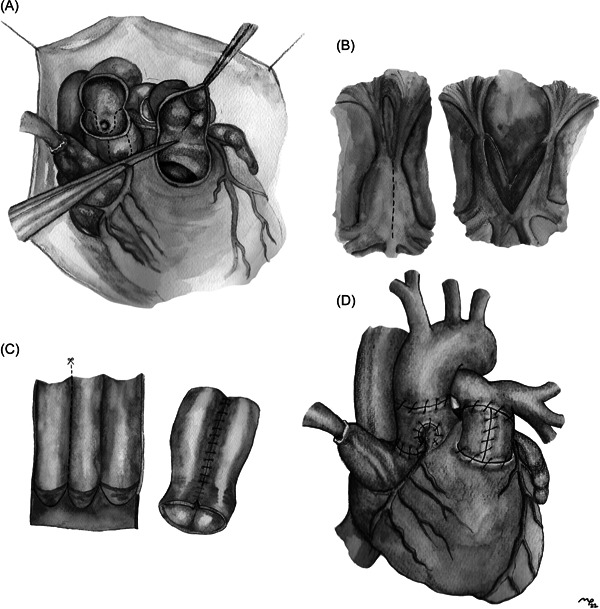
(A) Pulmonary autograft harvesting. (B) Papillary muscle splitting. (C) Contegra bicuspidalization. (D) Completed Ross produre

After 8 days the patient underwent a Ross procedure with mitral valve plasty and closure of the patent foramen ovale. The weight at the time of the operation was 2.100 kg and he was 2.3 months old (adjusted age for prematurity: 6 days of life). Cardiopulmonary bypass was established through a median sternotomy and aorto‐bicaval cannulation. One shot Del Nido Cardioplegia was administered at first in the aortic root (100 ml) and soon after selectively in the coronary ostia (30 and 20 ml in the left and right ostium, respectively). After the ascending aorta was opened, a detached aortic cusp was identified as a result of the previous balloon dilation. The pulmonary autograft was harvested and then implanted in the aortic position with a continuous 7‐0 polypropylene suture. Coronary buttons were reimplanted with 8‐0 polypropylene suture. After distal aortic suture, left atriotomy through Sondergaard's interatrial groove was performed in hypothermic circulatory arrest (22°C nasopharyngeal). The mitral valve leaflets were both dysplastic with an arch‐like subvalvular apparatus. A splitting of the papillary muscle was performed to increase leaflet mobility and augment their coaptation and closure of a patent foramen ovale was accomplished through a right atriotomy. Finally, a 12 mm Contegra Conduit was downsized to 8 mm (with a bicuspidalization according to the patient's body surface area) and then interposed between the right ventricle and the pulmonary artery with a continuous 7‐0 polypropylene suture (Figure 1). Cardiopulmonary bypass, crossclamp‐time and circulatory arrest time were 180, 123, and 4 min, respectively. Postoperative transesophageal echocardiogram showed good biventricular function, mild aortic regurgitation, and mild‐to‐moderate mitral regurgitation.

The postoperative course was noteworthy for inotropic support and temporary atrioventricular block. Chest closure was performed on the 2nd postoperative day and the patient was discharged on the 30th postoperative day. After 11 months the patient developed endocarditis of the Contegra conduit and subsequent stenosis with a maximum gradient of 90 mmHg (Video 2). Antibiotic treatment and stent placement in the proximal portion of the conduit were required with good results and without complications. At the last postoperative follow‐up at 38 months the patient was in optimal clinical conditions (weight 11.880 kg) and the last echocardiogram showed no dilation of the left ventricle with normal systolic function ejection fraction ( >70%), mild aortic regurgitation and mild‐to‐moderate mitral regurgitation (Video 3).

## 3 DISCUSSION

The Ross procedure is a very interesting option in children and young adults with severe aortic stenosis and regurgitation. Indeed, this operation not only allows for aortic valve replacement in a population where this option would not otherwise be possible, but also provides the potential for neoaortic valve growth.^4^

In this particular case, we performed this procedure as a rescue operation under emergent circumstances in a very low birth weight neonate. On this regard, it should also be emphasized that the net weight of the baby at the time of Ross operation would have been even lower considering his important edematous state.

**Figure 2:**
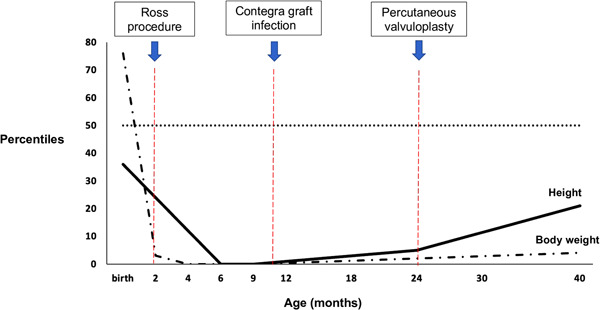
Height (continuous line) and body weight (dashdotted line) curves, expressed as percentiles for age

It should be considered that our patient presented with at least two important associated features (coarctation of the aorta and mitral valve dysplasia), placing him in an extremely high risk group.^5^ Moreover, given his worsening heart failure state it should be stressed that no other comparable therapeutic options were possible to save his life. Even though it was a risky operation, the results were outstanding both in terms of clinical outcomes and subsequent growth of the baby (Figure 2).

This case demonstrates a successful use of this procedure even in very small infants and it encourages its performance even in challenging circumstances.

## CONFLICTS OF INTEREST

The authors declare no conflicts of interest.

## ETHICS STATEMENT

IRB approval and informed consent were obtained.

## Supporting information

Video 1: Pre‐operative transthoracic echocardiogram showing in the parasternal long and short‐axis view a dilated left ventricle and a severely dysplastic and stenotic aortic valve. Apical four chamber view showing severe mitral regurgitation.

Video 2: 3D CT reconstruction before stent placement in the Contegra conduit.

Video 3: 38 months follow‐up transthoracic echocardiogram showing a well functioning neo‐aortic valve, only mild aortic regurgitation. 3D echo reconstruction of the mitral valve.
